# Transcriptome analysis of *Streptococcus gallolyticus* subsp. *gallolyticus* in interaction with THP-1 macrophage-like cells

**DOI:** 10.1371/journal.pone.0180044

**Published:** 2017-07-03

**Authors:** Imke Grimm, Nina Garben, Jens Dreier, Cornelius Knabbe, Tanja Vollmer

**Affiliations:** Institut für Laboratoriums- und Transfusionsmedizin, Herz- und Diabeteszentrum Nordrhein-Westfalen, Universitätsklinikum der Ruhr-Universität Bochum, Bad Oeynhausen, Germany; Oregon Health & Science University, UNITED STATES

## Abstract

**Background:**

*Streptococcus gallolyticus* subsp. *gallolyticus* (*S*. *gallolyticus*) is a pathogen of infective endocarditis. It was observed previously that this bacterium survives longer in macrophages than other species and the phagocytic uptake by and survival in THP-1 macrophages is strain-dependent.

**Methods:**

The phagocytosis assay was performed with THP-1 macrophages. *S*. *gallolyticus* specific whole genome microarrays were used for transcriptome analysis.

**Results:**

Better survival in macrophages was observed for UCN34, BAA-2069 and ATCC43143 than for DSM16831 and LMG17956. *S*. *gallolyticus* strains show high resistance to tested bactericidal agents (acid, lysozyme and hydrogen peroxide). *S*. *gallolyticus* stimulates significant lower cytokine gene expression and causes less lysis of macrophages compared to the control strain *Staphylococcus aureus*. *S*. *gallolyticus* reacts to oxidative burst with a higher gene expression of NADH oxidase initially at the early phase. Expression of genes involved in D-alanylation of teichoic acid, carbohydrate metabolism and transport systems were upregulated thereafter.

**Conclusion:**

*S*. *gallolyticus* is very resistant to bactericidal agents normally causing degradation of bacteria in phagolysosomes. Additionally, the D-alanylation of teichoic acid is an important factor for survival.

## Background

*Streptococcus gallolyticus* subsp. *gallolyticus* (previously *S*. *bovis* biotype I) is a Gram-positive human commensal which is also a pathogen of infective endocarditis (IE) [1]. Thereby, an association of IE caused by *Streptococcus gallolyticus* subsp. *gallolyticus* with colorectal neoplasms has often been described [2–5], but the pathomechanisms of this bacterium are still insufficiently understood [6–10].

Macrophages play a key role in innate immunity [11]. In the cardiac valve of patients with streptococcal-induced IE, 4.5% of the areas examined were covered by macrophages, whereas no macrophages were found in the cardiac valves without infection [12]. Boleij *et al*. have already shown that *S*. *gallolyticus* subsp. *gallolyticus* is able to survive much longer in macrophages than other bacteria (e.g. *Bacillus subtilis*) [13]. Macrophages are able to phagocyte pathogens and trap them in maturing phagosomes, killing them, breaking them down by bactericidal mechanisms and presenting their pathogens’ antigens on their surface [14,15]. Key features of phagocytosis are reactive oxygen species (ROS), nitrogen species, hydrolases and the decrease of the pH value [16]. Thereby, pathogenic bacteria have evolved mechanisms to evade killing by macrophages [17]. Some *Staphylococcus aureus* strains are able to survive the oxidative burst by different mechanisms or modify their peptidoglycan backbone to become lysozyme-resistant. *Streptococcus iniae* modulates the inflammatory response of macrophages for intracellular replication [18]. Herdt *et al*. observed that some *S*. *bovis* strains were able to evade phagosomes of macrophages from pigeons, replicated intracellularly and lysed all macrophages within 7 h [19].

In the present study, we analyzed the reaction of THP-1 macrophages to the phagocytosis of *S*. *gallolyticus* subsp. *gallolyticus* and vice versa the reaction of *S*. *gallolyticus* subsp. *gallolyticus* to THP-1 macrophages. Additionally, strain dependent survival in presence of different antimicrobial substances was revealed.

## Material and methods

### Cell culture and bacterial strains

THP-1 cells were cultivated in RPMI 1640 medium (RPMI 1640; Thermo Scientific, Waltham, USA) supplemented with 10% fetal calf serum (FCS; Pan Biotech; Aidenbach, Germany) and 1 × antibiotic/antimycotic solution (100 units/ml Penicillin, 0,1 mg/ml Streptomycin, 0,25 μg/ml Amphotericin B, AB/AM, Pan Biotech) at 37°C and 5% CO_2_. *S*. *gallolyticus* subsp. *gallolyticus* strains (Table 1) were grown overnight in brain heart infusion broth (BHI; Thermo Scientific, Waltham, USA) at 37°C and 220 rpm. Overnight cultures were used for phagocytosis assays. Bacterial cultures in the exponential growth phase were generated by inoculating 5 ml BHI medium with 100 μl overnight culture. The exponential growth phase was reached after 2.5 h at 37°C and 220 rpm. Cultures in the exponential phase were used to analyze the survival of bacteria in differently supplemented BHI. The bacterial titer was determined by serial dilutions in Dulbecco’s phosphate-buffered saline (DPBS) and plating 100 μl of an adequate concentration in triplicate on tryptone soya (TS) agar (Thermo Scientific, Waltham, USA). The TS agar plates were incubated at 37°C and the colonies that grew were counted by the aCOLyte colony counter (Synbiosis, Cambridge, Great Britain).

**Table 1:** List of bacterial strains used in this study with the source.

Species	Strain	Origin	source
*Streptococcus gallolyticus* subsp. *gallolyticus*	DSM16831	koala feces	DSMZ
*Streptococcus gallolyticus* subsp. *gallolyticus*	BAA-2069	human, IE patient	ATCC
*Streptococcus gallolyticus* subsp. *gallolyticus*	LMG17956	Bovine	LMG
*Streptococcus gallolyticus* subsp. *gallolyticus*	UCN34	human, IE patient	ATCC
*Streptococcus gallolyticus* subsp. *gallolyticus*	ATCC43143	human, blood	ATCC
*Staphylococcus aureus*	ATCC25923	human, clinical isolate	ATCC

DSMZ: German Collection of Microorganisms and Cell cultures; LMG: Laboratory of Microbiology, Ghent University; ATCC: American Type Culture Collection

### Phagocytosis assay

This phagocytosis assay was based on the assays of Boleji *et al*. and Kaneko *et al*. [13,20]. Either 1.5 × 10^6^ THP-1 monocytes per well were disseminated in 12-well plates or 1.3 × 10^5^ THP-1 monocytes per well were disseminated in 96-well plates with 50 ng/ml PMA-supplemented medium to differentiate the monocytes into macrophages within three days. On the third day, the medium was changed into PMA-free medium after washing the cells twice with DPBS (Thermo Scientific, Waltham, USA). An overnight culture of *S*. *gallolyticus* subsp. *gallolyticus* was serially diluted (10^3^ dilution) in RPMI 1640 supplemented with 10% FCS without AB/AM and the final bacterial titer of the inoculum was determined by plating assay. After washing the macrophages with DPBS three times, the *S*. *gallolyticus* subsp. *gallolyticus* dilution was added and plates were centrifuged at 400 × *g* for 5 min to assure the attachment of the bacteria to the macrophages (definition for time point -1 h). Phagocytic uptake of bacteria was ensured for 30 min at 37°C and 5% CO_2_ and the macrophages were washed thrice with DPBS. RPMI 1640 including 10% FCS, 1 × AB/AM and 200 μg/ml gentamycin was added for 20 min to kill the residual extracellular bacteria (definition for time point 0 h [13]). Saponin (1%; Sigma, Steinheim, Germany) lysed the macrophages at definition time point 0 h, 2.5 h, 5 h and 8 h the bacterial titer was determined by plating assay as described above. The rate of phagocytosis was defined as the percentage of bacteria at t = 0 h relative to the added inoculum of *S*. *gallolyticus* subsp. *gallolyticus*.

### Cytotoxicity assay

The cytotoxicity of different *S*. *gallolyticus* subsp. *gallolyticus* strains and *Staphylococcus aureus* on macrophages was measured using the commercial Pierce LDH Assay Kit (Thermo Scientific, Waltham, USA), following the manufacturer’s instructions. The macrophages were infected with *S*. *gallolyticus* subsp. *gallolyticus* or *Staphylococcus aureus* as described above. The plates were incubated for 5 h using a cell monolayer completely lysed by the lysis solution provided in the kit as a positive control. The negative control was determined by adding 10 μl water instead of bacteria. The plate was spun down at 250 × *g* for 3 min and 50 μl of the supernatants from infected cells, positive control and negative control were transferred to a new 96-well plate, 50 μl reaction mix was added to the supernatants and they were incubated for 30 min in the dark. The reaction was stopped by adding 50 μl stop solution. Absorption was measured by λ = 490 nm and λ = 680 nm. OD_680_ was measured to subtract the instrumental background.

The percentage of macrophages lysed was calculated by the following formula:

$$\mathrm{cytoxicity}=\frac{\mathrm{infected cells negative control}}{\mathrm{positive control - negative control}}\times 100=\mathrm{lysed cells}\;(\%)$$

Results of this assay are shown in Table 2 and Fig B2 in S1 File.

**Table 2:** Ratio of bacteria to macrophages in microscopic analysis and response of THP-1 macrophages to *Streptococcus gallolyticus* subsp. *gallolyticus* strains and *Staphylococcus aureus* ATCC 25923. 1+2) Microscopic analysis revealed the ratio of bacteria counted (without differentiation between dead and alive bacteria) to macrophages with the distinction between bacteria within and outside macrophages (bacteria/macrophage; counted macrophages are set as 1; Fig A and supplemental method in S1 File; n = 100 macrophages). 3) The oxidative reaction of macrophages to bacteria was detected by fluorescence signaling (DCF) at time point t = 0 h. H_2_O_2_ (1 mM) was used as stimulation control (Fig B1 in S1 File). 3) Cytotoxicity of bacteria to THP-1 macrophages compared to control (lysis of THP-1 macrophages by lysis buffer was set as 100%) was analyzed by LDH-assay 5 h after phagocytosis (Fig B2 in S1 File). 4–6) Gene expression of cytokines in THP-1 macrophages was determined by real-time PCR after stimulation with the different bacteria (t = 5 h); the control without bacterial stimulation was set as one (Fig C in S1 File). Results of statistical analysis (Mann-Whitney U test) of individual strains are arranged in tabular form. Only significant differences between strains and bacterial species are represented. Arbitrary units = au; SA = *Staphylococcus aureus*; bac = bacterial cells; I = intracellular; e = extracellular; m = macrophage; n = 3.

Analysis	Time point	DSM16831	BAA-2069	LMG17956	UCN34	ATCC43143	ATCC25923	Control
		S1	S2	S3	S4	S5	(*S*. *aureus*); SA	C
1) Location: intracellular bacteria per macrophage	**1 h**	**0.7**	**1.3**	**2.5**	**0.3**	**0.7**	**0.6**	**-**
2) Location: extracellular bacteria per macrophage	**1 h**	**0**	**0.03**	**0.004**	**0**	**0**	**0.8**	**-**
3) Oxidative burst(emission λ = 488 nm; au)	**0 h**	**645**	**557**	**625**	**603**	**722**	**683**	**328**
		** C	** C	** C	** C	** C	** C	** S1; S2; S3;
								S4; S5; SA
4) Cytotoxicity (%)	**5 h**	**18.0**	**21.9**	**17.9**	**17.8**	25.0	**33.4**	100
		* SA		* SA			* S1; S3	with lysis buffer
5) IL6 gene expression (au)	**5 h**	**2.5**	**4.2**	**3.0**	**1.5**	**3.3**	**32.6**	**1.0**
		** S4;	** S4;		** S1; S2		**** S1; S2; S3;	without stimulus
		**** SA	**** SA	**** SA	**** SA	**** SA	S4; S5	
6) IL8 gene expression (au)	**5 h**	**1.5**	**1.3**	**1.0**	**1.2**	**1.5**	**9.5**	**1.0**
		* S4; ** S3	** S3	* S4; ** S1; S2	* S1; S3; S5	* S4	**** S1; S2; S3;	without stimulus
		**** SA	**** SA	**** S5; SA	**** SA	**** S3; S5	S4; S5	
7) IL1B gene expression (au)	**5 h**	**1.4**	**1.3**	**1.1**	**0.9**	**1.2**	**5.8**	**1.0**
		* S4; ****SA	* S4; ****SA	****SA	* S5; **** SA	**** SA	**** S1; S2; S3; S4; S5	without stimulus

Results of statistical analysis between strains or control (Mann-Whitney U test)

*: *p <* 0.05

**: *p* < 0.005

****: *p* < 0.000

### Intracellular relative quantification of reactive oxidative species

For the quantification of reactive oxidative species, the phagocytosis assay was carried out as described above with 12 well plates and a MOI of five. The reactive oxidative species in macrophages were relatively quantified by dichlorofluorescin diacetate (DCFH-DA; Sigma, Steinheim, Germany). The DCFH-DA is hydrolyzed by intracellular esterases to DCFH. Reactive oxidative species oxidize DCFH, which results in a fluorescent signal. The macrophages were washed with DPBS and then incubated with RPMI 1640 medium supplemented with 10% FCS and 5 μM DCFH-DA for 30 min at 37°C and 5% CO_2_. Subsequently, the macrophages were washed three times to remove extracellular DCFH-DA. Overnight cultures were centrifuged and washed with DPBS to remove residual BHI medium. Macrophages including DCFH were infected with these cultures or incubated with H_2_O_2_ (1 mM). DCFH reacts with reactive oxygen species and becomes the fluorescent molecule DCF. Inititally after phagocytosis and incubation with antibiotic-supplemented medium (0 h) the emission of DCF was measured with the Tecan Infinite 200 PRO plate-reader (Männedorf, Switzerland) (t = 0 h) [21]. Fluorescence-excitation was carried out at λ = 488 nm and emission was detected at λ = 525 nm. The emission of the negative control (no stimulation) was subtracted from the emission of infected and H_2_O_2_-stimulated macrophages, respectively. Results of this assay are shown in Table 2 and Fig B1 in S1 File.

### Hydrogen peroxide sensitivity assay

Determination of the sensitivity of different *S*. *gallolyticus* subsp. *gallolyticus* strains to H_2_O_2_ stress was performed by the growth in BHI medium supplemented with different concentrations of H_2_O_2_ (Roth, Karlsruhe, Germany). All experiments were carried out in the dark. An amount of 1 ml BHI medium supplemented with H_2_O_2_ (0, 10, 15 or 20 mM) was inoculated with 20 μl exponential growth culture of different *S*. *gallolyticus* subsp. *gallolyticus* strains and *Staphylococcus aureus* for comparison. As control served the bacterial growth in BHI without any supplement. After 5 h of growth in these media, colony forming units (cfu) were determined by plating assay.

### Lysozyme resistance

Fresh BHI medium was inoculated to test the lysozyme resistance of different *S*. *gallolyticus* subsp. *gallolyticus* strains. Subsequently, 20 μl of bacterial culture (exponential phase) was added to 980 μl BHI medium in 24-well plates supplemented with lysozyme from chicken egg white (Sigma, Steinheim, Germany) (0, 5, 10 or 20 mg/ml), incubated at 37°C and 70 rpm. As control bacterial growth in BHI medium without any supplement was taken. After 5 h of growth in this medium, cfu were determined by plating assay as described above.

### Acid tolerance

Survival of *S*. *gallolyticus* subsp. *gallolyticus* strains at pH 4 was analyzed as follows. Bacterial cultures in the exponential phase were centrifuged (5,000 × *g*, 5 min) and the supernatant was discarded. The pellets were resuspended in BHI medium (pH = 7.4) or in BHI medium with a pH-value of 4 adjusted by hydrochloric acid. After 5 h of incubation at 37°C and 220 rpm, the cfu were determined by plating assay. The pH-value was checked after 5 h by pH indicator strips and no change in pH through inoculation was detected.

### RNA extraction of THP-1 macrophages with following cDNA synthesis

Isolation of RNA was performed with the NucleoSpin RNA II kit (Machery and Nagel, Düren, Germany), following the manufacturer’s instruction. The RNA was eluted with 30 μl RNase-free water and quantified using the NanoDrop 2000 (VWR, Radnor, USA). The synthesis of cDNA was carried out by using the Superscript II Reverse Transcriptase Kit (Invitrogen, Carlsbad, USA). An amount of 2 μg of RNA was diluted in 20 μl RNase-free water and cDNA was generated within three steps, following the manufacturer’s instructions. The cDNA was diluted with water at a ratio of 1:5 for real-time PCR.

### RNA extraction of *S*. *gallolyticus* subsp. *gallolyticus* with following cDNA synthesis for real-time PCR

Gene expression was analyzed from the two *S*. *gallolyticus* subsp. *gallolyticus* isolates BAA-2069 and UCN34. The RNA from these strains was isolated at three different time points. The initial time point was directly after centrifugation (400 × *g*, 5 min) of the 96-well plates (-1 h). The second RNA extraction was at the time point 0 h and the third at 5 h. The RNA was extracted with the peqGOLD Bacterial RNA Kit (VWR, Radnor, USA). Bacterial cells were resuspended in TE buffer and lysis buffer T and transferred to Lysing Matrix B tubes (MP Biomedicals, Santa Ana, USA). Bacterial cells were disrupted by using Vortex-Genie 2 (Scientific Industries, New York, USA) for 3 min at full speed. Further RNA extraction was carried out following the manufacturer’s instructions. The RNA was eluted with 30 μl RNase-free water and quantified using the NanoDrop 2000. RNA quality was determined with the Agilent RNA 6000 Pico Kit (Agilent). The RNA samples had a RIN value >8. The synthesis of cDNA was carried out with the High-Capacity cDNA Reverse Transcription Kit (Thermo Scientific, Waltham, USA), following the manufacturer’s instructions. The cDNA was generated from 500 ng RNA by a one-step PCR. The cDNA was diluted in water at a ratio of 1:10 for real-time PCR.

### Relative quantitative real-time PCR

Gene expression analysis of the cytokines in THP-1 macrophages and verification of *S*. *gallolyticus* subsp. *gallolyticus* microarray results were performed by real-time PCR using the LightCycler 480 (Roche, Basel, Switzerland). The reaction volume was 10 μl containing 2.5 μl cDNA, 0.25 μl each Primer (20 μM), 5.0 μl LightCycler 480 SYBR Green I Master-Kit and 2.0 μl water. The denaturation of the reaction mix took place initially at 95°C (10 min), followed by 45 cycles consisting of denaturation for 10 s at 95°C, annealing at 63 or 65°C, respectively (see Table A in S1 File), for 15 s and elongation at 72°C for 20 s. Additionally, a melting curve served as a control for the PCR amplification. Relative gene expression was calculated by normalizing with reference genes (human reference genes: ribosomal protein L13a, succinate dehydrogenase and hydroxymethylbilane synthase; bacterial reference genes: 16S rRNA and 23S rRNA) by the efficiency corrected ∆∆ct method [22]. These reference genes were determined by geNorm [22]. Intron-spanning primers were used for the human gene expression analysis. All oligonucleotides, their sequences and associated annealing temperatures are listed in Table A in S1 File.

### Gene expression analysis of *S*. *gallolyticus* subsp. *gallolyticus* using full-genome microarray

Phagocytosis assay was carried out as described above. Three biological replicates for each strain and time point were generated. To conclude false positive signals, RNA of THP-1 macrophages without bacteria was also processed and hybridized. Microarrays had a customized design (MyArray; OakLabs GmbH, Hennigsdorf, Germany) which was generated out of the sequences of four different *S*. *gallolyticus* subsp. *gallolyticus* genomes [7–9]. The microarrays were synthesized by Agilent and present 4,382 genes by 10,607 oligonucleotides consisting of 60 bases. The cDNA and cRNA synthesis, including Cy3-labeling, and the microarray hybridization was carried out with the Quick Amp WT Labeling Kit, one-color (Agilent, Santa Clara, USA), following the manufacturer’s recommendations, but with the following exceptions. Due to mixed RNA (prokaryotic and eukaryotic), more RNA (75 ng) was used for labeling and 900 ng labeled cRNA was used for hybridization. The quality of cRNA was proved by Nanodrop (Microarray Measurement; > 6 pmol Cy3 per μg cRNA) and Agilent Bioanalyzer (RNA signals from fragments with size 200–2000 nt) as specified by the manufacturer. Hybridization was extended to 38 h to achieve sufficient fluorescent signals. The slides were washed and hybridization was stabilized with Stabilization & Drying Solution (Agilent, Santa Clara, USA). After drying, the hybridized microarrays were scanned with the high-resolution Agilent microarray scanner G2565CA at a resolution of 5 μm and analyzed with the Feature extraction software (Agilent, Santa Clara, USA). Microarray data have been deposited in the Gene expression Omnibus (GEO) database at NCBI with GEO accession number GSE96865 at https://www.ncbi.nlm.nih.gov/geo/query/acc.cgi?acc=GSE96865.

### Microscopic analysis

The method of the microscopic analysis is described in the supplement in S1 File.

### Statistics

Experimental data were analyzed by Mann-Whitney U test using GraphPad Prism 6.0 (GraphPad Software). P values less than 0.05 were considered as statistically significant. Mean with standard error is displayed in figures. For microarray analysis raw data were quantile-normalized, statistical t-test (*Welch’s t-test*) gene expression data were generated by the Direct Array software (OakLabs, Hennigsdorf, Germany). Thereby, all log_2_ values between -1 and 1 were ignored and only statistically significant values (*p* < 0.05) are displayed.

## Results

### Phagocytosis of five *S*. *gallolyticus* subsp. *gallolyticus* strains

**Fig 1:**
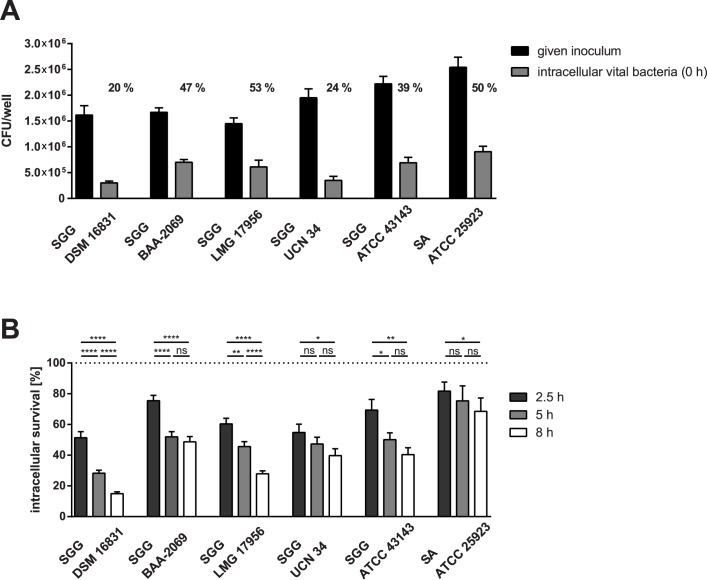
Phagocytic uptake of *S*. *gallolyticus* subsp. *gallolyticus* strains through THP-1 macrophages and survival of these within THP-1 macrophages. (A) Inoculum given (black bars) compared to the internalized number of *S*. *gallolyticus* subsp. *gallolyticus* strains by THP-1 macrophages (grey bars; t = 0 h). Additionally, the percentage of the phagocytic uptake of the given inoculum is shown; n = 4. (B) Percentage survival of *S*. *gallolyticus* subsp. *gallolyticus* in THP-1 macrophages after 2.5, 5 and 8 h of incubation compared to the internalized number of bacteria at time point t = 0 h (which is set as 100%; see dotted line). Statistical significance between the different time points of a strain is marked with stars obtained from the Prism Mann-Whitney U test; statistical results between strains are shown in Table B in S1 File. *: *p <* 0.05; **: *p* < 0.005; ****: *p* < 0.0001; n = 4. The standard error is marked with error bars. SGG = *Streptococcus gallolyticus* subsp. *gallolyticus;* SA = *Staphylococcus aureus*.

Five *S*. *gallolyticus* subsp. *gallolyticus* strains were analyzed in comparison to *S*. *aureus* ATCC25923 as control in all experiments. The differences in the phagocytic uptake of the different strains and the intracellular survival within 8 h are shown in Fig 1. Only about 20% of the inoculum of the strains DSM16831 and UCN34 given was taken up through phagocytosis by THP-1 macrophages. More bacterial cells of LMG17956 and BAA-2069 and the *S*. *aureus* strain ATCC25923 were phagocytized (about 50%), whereas 39% of the inoculum of the strain ATCC43143 given was found intracellularly in THP-1 macrophages. After eight hours of incubation the two strains DSM16831 and LMG17956 were killed significantly more compared to other strains (Fig 1B). Microscopic analysis revealed how many bacterial cells per macrophage are present at t = 1 h (Table 2; row: 2–3). Thereby, distinction between live and dead cells was not possible. It was observed that the *S*. *gallolyticus* subsp. *gallolyticus* strains were generally located intracellularly, whereas the *S*. *aureus* strain ATCC25923 has been found extracellular of THP-1 macrophages (Fig A in S1 File; supplementary methods).

The following aspects of the macrophages’ response to bacteria were analyzed to determine the source of these strain-dependent differences by the survival within THP-1 macrophages: 1) oxidative burst (row: 4), Table 2) cytokine expression of macrophages (rows: 6–8) and 3) cell lysis (row: 5). The THP-1 macrophages developed the same oxidative burst after stimulation with *S*. *gallolyticus* subsp. *gallolyticus* or *S*. *aureus*. Lysis of the macrophages did not differ between the *S*. *gallolyticus* subsp. *gallolyticus* strains after 5 h of stimulation. Indeed, cell lysis was significantly lower compared to the lysis caused by *S*. *aureus* (p < 0.05; Table 2). After 5 h of stimulation with *S*. *gallolyticus* subsp. *gallolyticus*, the gene expression of *IL8* and *IL-1B* was not considerably increased. Thereby, gene expression of all three cytokines increased much more highly through phagocytosis of the *S*. *aureus* strain ATCC25923 (Table 2).

### Survival and growth of *S*. *gallolyticus* subsp. *gallolyticus* under different conditions

**Fig 2:**
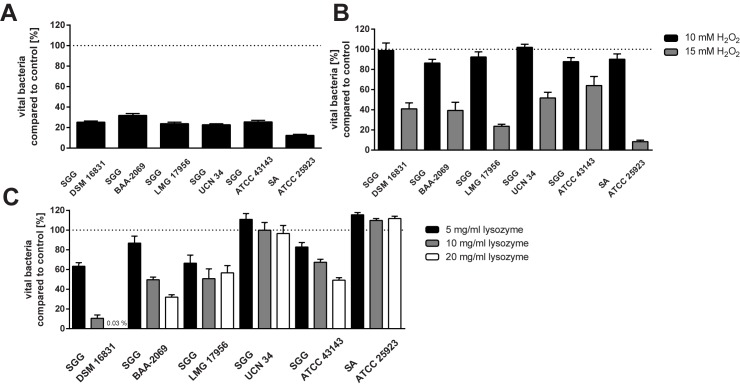
Growth or survival of *S*. *gallolyticus* subsp. *gallolyticus* under different conditions. (A) Survival of *S*. *gallolyticus* subsp. *gallolyticus* in BHI at pH 4 compared to a neutral environment which was set as 100% (dotted line) after 5 h incubation at 37°C. (B) Growth of *S*. *gallolyticus* subsp. *gallolyticus* in medium supplemented with different hydrogen peroxide (H_2_O_2_) concentrations (10 and 15 mM) compared to growth without H_2_O_2_, which was set as 100% (dotted line) after 5 h incubation at 37°C. (C) Growth of *S*. *gallolyticus* subsp. *gallolyticus* in lysozyme-supplemented BHI medium (5, 10, 20 mg/ml lysozyme) compared to growth without lysozyme which was set as 100% (dotted line) after 5 h incubation at 37°C. n = 3; mean with standard error is shown. SGG = *Streptococcus gallolyticus* subsp. *gallolyticus;* SA = *Staphylococcus aureus*.

Specific characteristics of bacteria can influence their death or survival in macrophages, such as resistances to reactive oxygen species, acid or lysozyme. Therefore, we examined strain-dependent differences under these different bactericidal conditions (Fig 2). All Figs show the percentage of vital bacteria in the presence of the bactericidal agent compared to the respective control (100%). It was shown that about 25% of the *S*. *gallolyticus* subsp. *gallolyticus* cells survived in acid medium (pH 4). The isolate BAA-2069 survived slightly better (32%; p < 0.05) than the other *S*. *gallolyticus* subsp. *gallolyticus* isolates. The *S*. *aureus* isolate ATCC25923 was significantly more susceptible to the acid medium than the *S*. *gallolyticus* subsp. *gallolyticus* isolates (p < 0.0005; Fig 2A).

Resistance to H_2_O_2_ was tested in BHI medium with different concentrations of this reactive oxygen agent (Fig 2B). A concentration of 15 mM led to a survival of 24% for the isolate LMG17956. Altogether, the five *S*. *gallolyticus* subsp. *gallolyticus* isolates analyzed were more resistant to 15 mM H_2_O_2_ than the *S*. *aureus* isolate ATCC25923 tested (Fig 2B; p < 0.0001), which showed only viable bacteria of 8% compared to the control. No living bacterial cells were detected in medium supplemented with 20 mM H_2_O_2_.

Furthermore, *S*. *gallolyticus* subsp. *gallolyticus* showed strain-dependent growth in the presence of different lysozyme concentrations (Fig 2C). The isolate UCN34 survived as well as the *S*. *aureus* isolate ATCC25923 in the presence of lysozyme concentrations up to 20 mg/ml. These strains showed even an increase in growth, UCN 34 with 5 mg/ml and the *S*. *aureus* control strain with any lysozyme supplement (5 mg/ml; 10 mg/ml; 20 mg/ml). By contrast, the growth ability of DSM16831 was highly impaired: after 5 h of incubation, only 0.03% vital bacterial cells were found compared to the control. The growth ability of BAA-2069 and ATCC43143 decreased with increasing lysozyme concentration from about 80% (5 mg/ml lysozyme) to 32% or 49%, respectively (20 mg/ml). Lysozyme impaired the growth of the isolate LMG17956 to 40–50%.

### Gene expression analysis of *S*. *gallolyticus* subsp. *gallolyticus* using a full genome microarray

**Fig 3:**
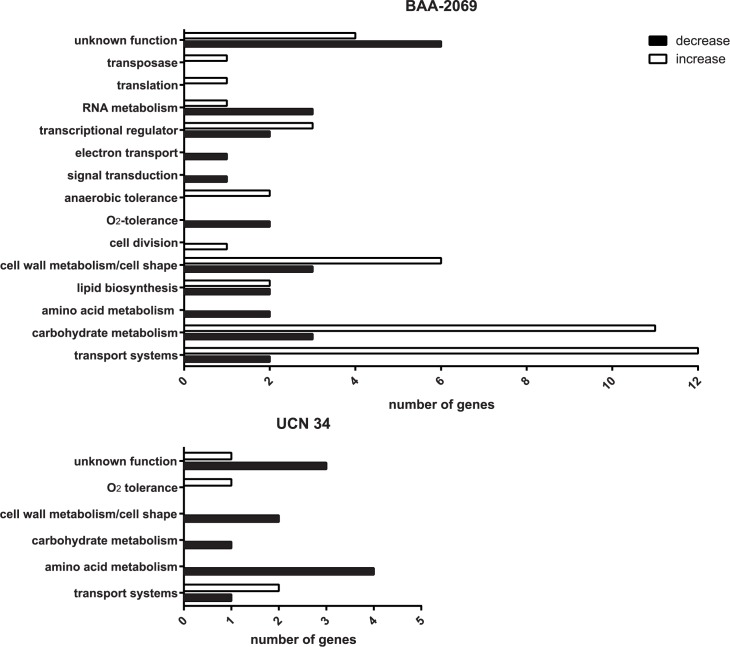
The number of regulated genes found by microarray analysis. The genes whose expression were increased (white) or decreased (black) after phagocytosis (0 h and 5 h together) compared to -1 h (attachment of bacteria) are represented. The number of genes which were regulated in the *S*. *gallolyticus* subsp. *gallolyticus* strains BAA-2069 and UCN34 through phagocytic uptake by THP-1 macrophages are displayed on the x-axis. The functional categories in which the genes are sorted are displayed on the y-axis.

The isolates BAA-2069 and UCN34 were used for microarray experiments because they have shown a better survival in THP-1 macrophages. The gene expression is normed on the time point -1 h, directly after bacteria were attached to the macrophages through centrifugation. The gene expression was normalized to this time point (-1 h), which is set as 0 (log2 ratio) or 1 (fold change). Changes in gene expression are shown for the time points 0 h (20 min incubation with antibiotic-supplemented medium) and 5 h later. Changes between 0 h and 5 h do not differ from the data presented (-1 h and 5 h) and are therefore not displayed. Microarray results, sorted by function, including the log2 ratio, the fold change and the corresponding p-value are shown in Table 3. Microarray analysis revealed 70 differentially regulated genes in BAA-2069, whereas only 15 genes were detected in UCN34 (Fig 3). According to the microarray data, most genes in BAA-2069 were regulated after 5 h of incubation and only a few genes were regulated prior at 0 h (Table 3). Regulated genes belong mainly to transport systems, such as the phosphotransferase (PTS) system transporter and ATP transporter, and the metabolisms of carbohydrates, for example, the *glg* (*glgBCDP*) operon and the *dlt* (*dltABCD*) operon, whose products influence the cell wall. At the earlier time point (0 h), gene expression was more regulated in the *S*. *gallolyticus* subsp. *gallolyticus* strain UCN34 compared to BAA-2069. Similar to BAA-2069, genes of the PTS system transporter are upregulated, but more genes are downregulated, especially genes of the amino acid metabolism.

**Table 3:** Differentially regulated genes of *S*. *gallolyticus* subsp. *gallolyticus* BAA-2069 and UCN34 through phagocytosis by THP-1 macrophages in microarray analysis sorted by function. Changes in gene expression after phagocytosis at time point 0 h and 5 h are shown compared to the transcriptome at time point -1 h (attachment to macrophages). Fluorescence intensities of the microarrays were quantile normalized and computed log2 values and fold changes with significances (Welch’s test) are listed; n = 3; n. r. = not regulated.

**BAA-2069: Increased gene expression**			**log2Ratio**		**p-value**		**foldchange**	
**function**	**gene**	**protein**	**0 h**	**5 h**	**0 h**	**5 h**	**0 h**	**5 h**
**carbohydrate metabolism**	*lacC*	tagatose 6-phosphate kinase	1.07	n. r.	0.02	-	2.10	n. r.
	*pflB*	formate acetyltransferase	n. r.	1.19	-	0.04	n. r.	2.28
	*malM*	4-alpha-glucanotransferase	n. r.	1.34	-	0.004	n. r.	2.53
	*treA*	trehalose-6-phosphate hydrolase	n. r.	1.37	-	0.03	n. r.	2.58
	*yjbF*	SNARE-like family protein	n. r.	1.42	-	0.02	n. r.	2.68
	*SGGBAA2069_c06730*	putative CoA-substrate-specific enzyme activase	n. r.	1.51	-	0.01	n. r.	2.85
	*glgP*	starch phosphorylase	n. r.	1.55	-	0.003	n. r.	2.93
	*glgC*	glucose-1-phosphate adenylyltransferase	n. r.	1.72	-	0.02	n. r.	3.29
	*nagA*	N-acetylglucosamine-6-phosphate deacetylase	n. r.	1.74	-	0.02	n. r.	3.34
	*glgD*	glucose-1-phosphate adenylyltransferase	n. r.	1.85	-	0.01	n. r.	3.61
	*glgB*	glycogen branching protein	n. r.	2.1	-	0.05	n. r.	4.29
**transport system**	*malF*	maltodextrin transport system permease malF	n. r.	1.05	-	0.04	n. r.	2.07
	*atpC*	F0F1 ATP synthase subunit epsilon	n. r.	1.37	-	0.02	n. r.	2.58
	*fatA*	iron complex transport system ATP-binding protein	n. r.	1.61	-	0.05	n. r.	3.05
	*msmK*	Multiple sugar-binding transport ATP-binding protein	n. r.	2.13	-	0.04	n. r.	4.38
	*mtsC*	metal cation ABC transporter membrane protein	n. r.	1.92	-	0.05	n. r.	3.78
	*manN*	PTS system mannose-specific transporter subunit IID	n. r.	2.19	-	0.05	n. r.	4.56
	*manL*	PTS system mannose-specific transporter subunit IIA	n. r.	2.21	-	0.05	n. r.	4.63
	*adcA*	Zinc-binding protein adcA	n. r.	2.23	-	0.05	n. r.	4.69
	*manM*	PTS system mannose-specific transporter subunit IIC	n. r.	2.32	-	0.03	n. r.	4.99
	*treB*	PTS system trehalose-specific transporter subunit IIA	n. r.	2.59	-	0.01	n. r.	6.02
	*SGGBAA2069_c02000*	PTS system galactitol-specific transporter subunit IIB	1.18	n. r.	0.01	-	2.27	n. r.
**transcriptional regulator**	*SGGBAA2069_c18600*	putative transcriptional regulator	1.53	1.2	0.03	0.05	2.89	2.30
	*SGGBAA2069_c7250*	LacI family transcriptional regulator	n. r.	1.52	-	0.02	n. r.	2.87
	*SGGBAA2069_c10720*	transcriptional regulator	1.03	n. r.	0.03	-	2.04	n. r.
**lipid biosynthesis**	*fabD*	malonyl CoA-acyl carrier protein transacylase	n. r.	1.41	-	0.02	n. r.	2.66
	*accD*	acetyl-CoA carboxylase subunit alpha	n. r.	1.77	-	0.04	n. r.	3.41
**anaerobic tolerance**	*nrdD*	anaerobic ribonucleoside triphosphate reductase	n. r.	1.5	-	0.04	n. r.	2.83
	*adhE*	acetaldehyde dehydrogenase	n. r.	2.08	-	0.01	n. r.	4.23
**cell wall metabolism/cell shape**	*SGGBAA2069_c13580*	autolysin	n. r.	1.55	-	0.003	n. r.	2.93
	*lss*	N-acetylmuramidase/lysin	n. r.	2.25	-	0.01	n. r.	4.76
	*dltA*	D-alanine—poly(phosphoribitol) ligase subunit 1	n. r.	3.8	-	0.04	n. r.	13.93
	*dltD*	D-alanine extramembranal transfer protein	n. r.	3.03	-	0.04	n. r.	8.17
	*dltC*	D-alanine—poly(phosphoribitol) ligase subunit 2	n. r.	3.29	-	0.03	n. r.	9.78
	*dltB*	D-alanine transfer protein DltB	n. r.	3.56	-	0.03	n. r.	11.79
**NTP synthesis**	*ndk*	nucleoside diphosphate kinase	1	-	0.03	-	2.00	-
**translation**	*rpsU*	30S ribosomal protein S21	1.23	-	0.04	-	2.35	-
**transposase**	*SGGBAA2069_c02540*	transposase OrfB	1.76	-	0.05	-	3.39	-
**unknown function**	*SGGBAA2069_c21480*	conserved hypothetical protein	n. r.	1.21	-	0.002	n. r.	2.31
	*SGGBAA2069_c19440*	putative secreted protein	n. r.	1.22	-	0.02	n. r.	2.33
	*SGGBAA2069_C20110*	hypothetical protein	n. r.	2.3	-	0.04	n. r.	4.92
	*SGGBAA2069_c01150*	predicted membrane protein	n. r.	2.92	-	0.04	n. r.	7.57
**BAA-2069: Decreased gene expression**			**log2Ratio**		**p-value**		**foldchange**	
**function**	**gene**	**protein**	**0 h**	**5 h**	**0 h**	**5 h**	**0 h**	**5 h**
**transcriptional regulator**	*SGGBAA2069_c22710*	MarR family transcriptional regulator	n. r.	-2.51	-	0.03	n. r.	0.18
	*SGGBAA2069_c18000*	BadM/Rrf2 family transcriptional regulator	n. r.	-1.27	-	0.03	n. r.	0.41
**transport systems**	*SGGBAA2069_c04070*	polar amino acid transport system substrate-binding protein	n. r.	-2.46	-	0.05	n. r.	0.18
	*SGGBAA2069_c04080*	amino acid ABC transporter membrane protein	n. r.	-1.83	-	0.05	n. r.	0.28
**RNA metabolism**	*deaD*	DEAD/DEAH box helicase	n. r.	-2.33	-	0.05	n. r.	0.20
	*obgE*	GTP-binding protein, GTP1/Obg family	n. r.	-1.76	-	0.04	n. r.	0.30
	*spoU*	RNA methyltransferase, TrmH family	n. r.	-1.16	-	0.03	n. r.	0.45
**carbohydrat metabolism**	*SGGBAA2069_c14130*	putative glyoxalase/bleomycin resistance protein/dioxygenase	n. r.	-2.22	-	0.01	n. r.	0.21
	*SGGBAA2069_c20810*	carbonic anhydrase	n. r.	-1.45	-	0.03	n. r.	0.37
	*icd*	isocitrate dehydrogenase	n. r.	-1.23	-	0.03	n. r.	0.43
**cell wall metabolism/cell shape**	*prsA1*	Parvulin-like peptidyl-prolyl isomerase	n. r.	-1.75	-	0.03	n. r.	0.30
	*SGGBAA2069_c12330*	polysaccharide deacetylase	n. r.	-1.13	-	0.03	n. r.	0.46
	*mreC*	Rod shape-determining protein	n. r.	-1.08	-	0.03	n. r.	0.47
**amino acid metabolism**	*hipO1*	aminoacylase/N-acyl-L-amino acid amidohydrolase/hippurate hydrolase	n. r.	-1.7	-	0.02	n. r.	0.31
	*lysA*	diaminopimelate decarboxylase	n. r.	-1.31	-	0.05	n. r.	0.40
**oxygen tolerance**	*nrdH*	glutaredoxin-like protein	n. r.	-1.34	-	0.04	n. r.	0.40
	*nrdE*	ribonucleotide-diphosphate reductase subunit alpha	n. r.	-1.05	-	0.04	n. r.	0.48
**lipid biosynthesis**	*ysfG*	diacylglycerol kinase	n. r.	-1.32	-	0.03	n. r.	0.40
	*plsX*	glycerol-3-phosphate acyltransferase	n. r.	-1.27	-	0.02	n. r.	0.41
**cell division**	*parB*	chromosome partitioning protein	n. r.	-1.17	-	0.05	n. r.	0.44
**signal transduction**	*acp1*	protein-tyrosine phosphatase	n. r.	-1.09	-	0.04	n. r.	0.47
**electron transport**	*yqiG*	NADH:flavin oxidoreductase	-1.28	n. r.	0.03	n. r.	0.41	-
**unknown function**	*SGGBAA2069_ c04900*	ubiquitin-binding YukD-like protein	n. r.	-2.56	-	0.03	n. r.	0.17
	*SGGBAA2069_c04760*	lipoprotein	n. r.	-2.24	-	0.04	n. r.	0.21
	*yukB*	FtsK/SpoIIIE family protein	n. r.	-1.91	-	0.01	n. r.	0.27
	*SGGBAA2069_c17200*	hypothetical protein	n. r.	-1.82	-	0.05	n. r.	0.28
	*SGGBAA2069_c11130*	hypothetical protein	n. r.	-1.47	-	0.04	n. r.	0.36
	*SGGBAA2069_c00040*	hypothetical protein	n. r.	-1.39	-	0.03	n. r.	0.38
**UCN34: Increased gene expression**			**log2Ratio**		**p-value**		**foldchange**	
**function**	**gene**	**protein**	**0 h**	**5 h**	**0 h**	**5 h**	**0 h**	**5 h**
**transport systems**	*levA/GALLO_0117*	PTS system N-acetylgalactosamine-specific transporter subunit IIA	2.2	n. r.	0.02		4.59	n. r.
	*GALLO_0118*	PTS system mannose-specific transporter subunit IIB	1.99	n. r.	0.02		3.97	n. r.
**unknown function**	*GALLO_2058*	hypothetical protein (CsbD-like protein)	1.44	n. r.	0.04		2.71	n. r.
**oxygen tolerance**	*nox*	NADH oxidase	1.4	n. r.	0.03		5.64	n. r.
**UCN34: Decreased gene expression**			**log2Ratio**		**p-value**		**foldchange**	
**function**	**gene**	**protein**	**0 h**	**5 h**	**0 h**	**5 h**	**0 h**	**5 h**
**metabolism (amino acid)**	*dapD*	2,3,4,5-tetrahydropyridine-2,6-carboxylate N-succinyltransferase	-1.36	-1.97	0.02	0.03	0.39	0.26
	*asd*	aspartate-semialdehyde dehydrogenase	n. r.	-1.03		0.01	n. r.	0.49
	*GALLO_0809*	GTP-binding protein	n. r.	-1.03		0.03	n. r.	0.49
	*ilvA*	threonine dehydratase	-1.54	n. r.	0.03		0.34	n. r.
**Unknown function**	*GALLO_0901*	hypothetical protein	n. r.	-1.63		0.04	n. r.	0.32
	*GALLO_0814*	hypothetical protein	n. r.	-1.25		0.04	n. r.	0.42
	*GALLO_1321*	glutamate-rich protein GrpB	-1.39	n. r.	0.01		0.38	n. r.
**transport system**	*GALLO_1556*	amino acid ABC transporter substrate-binding protein	-1.89	n. r.	0.03		0.27	n. r.
**cell wall metabolism/cell shape**	*GALLO_1358*	polyglycerol phosphate synthase	-1.76	n. r.	0.004		0.30	n. r.
	*gcaD*	UDP-N-acetylglucosamine pyrophosphorylase	-1.04	n. r.	0.01		0.49	n. r.
**metabolism (carbohydrate)**	*drm*	phosphopentomutase	-1.02	n. r.	0.05		0.49	n. r.

### Verification of microarray analysis by real-time PCR

**Fig 4:**
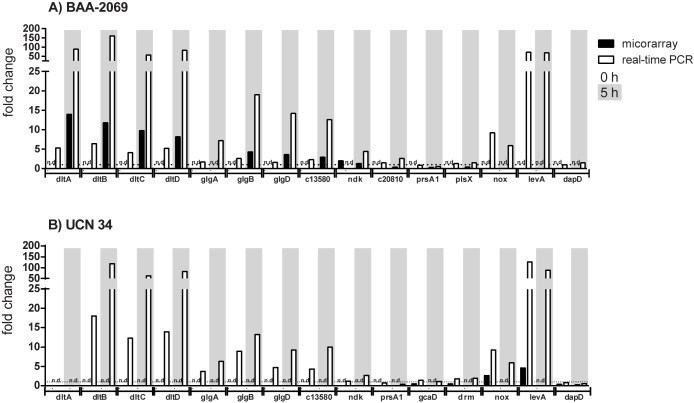
Validation of gene expression changes determined by microarray analysis through relative quantitative real-time PCR. The fold change of the regulation of distinct genes (x-axis) identified by microarray analysis (black) and real-time PCR (white) are represented. Bars with white background represent changes in gene expression at time point 0 h and bars with grey background represent changes in gene expression at time point 5 h, both are normalized on time point -1 h; n.d. = not detected.

Changes in gene expression were further analyzed by performing relative quantitative real-time PCR with more replicates to confirm the microarray results. Representative genes were selected from especially interesting ones. These genes were analyzed in both *S*. *gallolyticus* subsp. *gallolyticus* strains BAA-2069 and UCN34 to detect strain-dependent differences (Fig 4). It was shown that the genes of the *dlt* and *glg* operon were highly expressed in both isolates after 5 h compared to the control (-1 h). Changes of expression of the genes of the *dlt* and the *glg* operon which are found by microarray analysis in BAA-2069 were also found in UCN34 by relative quantitative real-time PCR. Additionally, the changes in gene expression of *nox* (NADH oxidase), which was found in UCN34 at time point 0 h, was also found in BAA-2069 by real-time PCR at this time point. Changes in gene expression were detected by real-time PCR much more highly, through the higher sensitivity of this method and another normalization method compared to the analysis by whole genome microarray. Downregulated genes found by microarray analysis often showed a less strong change in gene expression in real-time PCR experiments (Fig 4).

## Discussion

Circulating *S*. *gallolyticus* subsp. *gallolyticus* cells in the host must be able to survive human defense mechanisms, such as phagocytosis, to colonize at the human endocardium. Therefore, we analyzed the interaction of *S*. *gallolyticus* subsp. *gallolyticus* with THP-1 macrophages. The THP-1 cell line was obtained from a one-year old boy with monocytic leukemia [23]. PMA-differentiated THP-1 monocytes are often used in studies to analyze the macrophage-mediated phagocytosis of different pathogens [24–27]. In contrast to primary isolated monocytes, which go without stimuli into apoptosis, THP-1 monocytes are able to grow *in vitro* [28]. Therefore, the experiments were always performed under the same conditions without the influence of individual host factors. We focused on strain-dependencies and the gene regulation of *S*. *gallolyticus* subsp. *gallolyticus* by binding (-1 h), internalization and first reaction to macrophages (0 h), and the survival within phagolysosomes (5 h).

Boleij *et al*. revealed that *S*. *gallolyticus* subsp. *gallolyticus* is able to survive longer in THP-1 macrophages than *Bacillus subtilis* or *Lactobacillus plantarum* [13]. We observed that the phagocytic uptake and the survival of *S*. *gallolyticus* subsp. *gallolyticus* in macrophages depends on the isolate. The strains BAA-2069 and UCN34, for example, showed a persistent titer in macrophages within a distinct time period (I. Grimm, M. Weinstock, I. Birschmann, J. Dreier, C. Knabbe, and T. Vollmer, submitted). This study revealed that *S*. *gallolyticus* subsp. *gallolyticus* was not able to lyse macrophages actively, and hardly any differences in stimulation of the oxidative burst and cytokine gene expression were observed. Cytokine gene expression was induced by the *S*. *aureus* strain ATCC25923 significantly more highly. A potential explanation might be that *S*. *aureus* stimulates the cytokine expression higher through the lysis of macrophages, another that *S*. *gallolyticus* subsp. *gallolyticus* has mechanisms to alter the inflammatory response [29].

Phagolysosomes have different bactericidal mechanisms to kill and degrade microorganisms. We analyzed three of these to examine how resistant *S*. *gallolyticus* subsp. *gallolyticus* is and whether the resistance can influence its survival within macrophages. It was shown that *S*. *gallolyticus* subsp. *gallolyticus* survives better in an acidic environment than *S*. *aureus*, which could be an advantage in survival in phagolysosomes [17,30]. According to the microarray results, protection against reactive oxygen species is enabled by higher expression of *nox*. It is known that no more than 30 μM H_2_O_2_ would accumulate in phagosomes in addition to other reactive oxygen species [31]. Therefore, all the strains tested in this study would survive the bactericidal effectivity of H_2_O_2_ of phagosomes. Thereby, lysozyme resistance can support the establishment of an infection by, for example, better survival in phagocytes [32]. It was shown for *Streptococcus bovis* that lysozyme enables differentiation between human and bovine isolates because human isolates were more resistant to lysozyme [33]. We could not confirm these distinctions between the *S*. *gallolyticus* subsp. *gallolyticus* strains in this study. Although *S*. *gallolyticus* subsp. *gallolyticus* is a lysozyme-resistant bacterial species, no known gene for peptidoglycan modification was found in these *S*. *gallolyticus* subsp. *gallolyticus* strains by in silico analysis [6–9,32]. Interestingly, we recognized an increase of bacterial growth of the *S*. *gallolyticus* subsp. *gallolyticus* strain UCN34 and the *S*. *aureus* strain ATCC 25923 under different concentrations of lysozyme. This phenomenon has not been recognized for bacteria yet, however at the moment an explanation would be too speculative, since to our knowledge there is no literature providing supplying explanations regarding this aspect. It was shown overall that *S*. *gallolyticus* subsp. *gallolyticus* is quite resistant to bactericidal agents which allow killing and degrading pathogens in phagolysosomes. This could explain how *S*. *gallolyticus* subsp. *gallolyticus* is able to survive longer in THP-1 macrophages than *Bacillus subtilis* and *Lactobacillus plantarum* [13]. Additionally, the more rapid reduction of vital cells of the isolates DSM16831 and LMG17956 from animals may be explained by the susceptibility of DSM16831 to lysozyme and the susceptibility of LMG17956 to H_2_O_2_.

The isolate DSM16831 presented a remarkable phenotype which showed less virulent characteristics compared to the other strains tested. This phenotype was further confirmed in this study, since the *S*. *gallolyticus* subsp. *gallolyticus* strain DSM16831 was constantly killed in THP-1 macrophages and was less lysozyme-resistant compared to the other strains ([10], I. Grimm, M. Weinstock, I. Birschmann, J. Dreier, C. Knabbe, and T. Vollmer, submitted).

This is the first study using a full genome microarray to analyze the gene expression of *S*. *gallolyticus* subsp. *gallolyticus*. The first reaction (0 h) to phagocytic uptake is the reaction to the oxidative burst by expressing *nox*, which is a ROS-metabolizing enzyme [34]. Later (5 h), *S*. *gallolyticus* subsp. *gallolyticus* changes mainly the gene expression of proteins which are involved in cell shape/cell wall synthesis and different metabolisms, such as carbohydrates and lipids. The highest change in gene expression in the *S*. *gallolyticus* subsp. *gallolyticus* BAA-2069 and UCN34 through phagocytotic uptake was found in the *dlt* operon. The products of this operon are essential for the D-alanine substitution of teichoic acids (TAs), which leads to a resistance to cationic antimicrobial peptides [35]. It was shown for *Streptococcus agalactiae* that deficiency in D-alanyl-TA leads to a higher susceptibility in phagocytes and contributes to virulence [36]. This could be one of the virulence factors of *S*. *gallolyticus* subsp. *gallolyticus* to survive bactericidal agents in phagolysosomes. Another highly expressed gene after phagocytosis was *levA*, in addition to other genes which belong to PTS-systems. These transport systems are synthesized when the preferred carbon source is exhausted, which could be explained through the phagocytosis from nutrient-rich medium into phagosomes [37]. Another interesting regulated gene is *SGGBAA2069_c13580*, which codes for an autolysin. The in silico analysis of this gene showed similarities to *atlA* of *Streptococcus mutans*. It was observed that the product of this gene increased fibronectin-binding and contributed to bacterial survival in blood and resistance to phagocytosis; therefore, it was hypothesized that AtlA is a virulence factor in IE [38].

In conclusion, it was shown in the present study that *S*. *gallolyticus* subsp. *gallolyticus* is located intracellularly and hardly stimulates cytokine gene expression in THP-1 macrophages without lysing these cells. *S*. *gallolyticus* subsp. *gallolyticus* is resistant to most bactericidal agents which are characteristic for phagolysosomes. Microarray results revealed that D-alanylation of TA is an important factor for survival. Therefore, we conclude that *S*. *gallolyticus* subsp. *gallolyticus* survives in macrophages through changes in the cell wall of this bacterium.

## Supporting information

S1 File Supplement File S1: Supplementary methods, figures (A-C) and tables (A+B).
(DOCX)

## Abbrevations

AB/AM: antibiotic/antimycotic solution
BHI: brain heart infusion broth
cfu: colony forming units
DCFH-DA: dichlorofluorescin diacetate
DPBS: Dulbecco’s phosphate-buffered saline
FCS: fetal calf serum
H_2_O_2_: hydrogen peroxide
IE: infective endocarditis
IL: interleukin
MOI: multiplicity of infection
TA: teichoic acid

## References

[1] Olmos C, Vilacosta I, Sarriá C, López J, Ferrera C, Sáez C, et al (2016) *Streptococcus bovis* endocarditis: Update from a multicenter registry. American heart journal 171: 7–13. doi: 10.1016/j.ahj.2015.10.012 26699595 10.1016/j.ahj.2015.10.012

[2] Abeni C, Rota L, Ogliosi C, Bertocchi P, Centurini PB, Zaniboni A (2013) Correlation among *Streptococcus bovis*, endocarditis and septicemia in a patient with advanced colon cancer: a case report. J Med Case Rep 7: 185 doi: 10.1186/1752-1947-7-185 23855909 10.1186/1752-1947-7-185 PMC3750266

[3] Darjee R, Gibb AP (1993) Serological investigation into the association between *Streptococcus bovis* and colonic cancer. J Clin Pathol 46: 1116–1119. 8282836 10.1136/jcp.46.12.1116 PMC501723

[4] Lazarovitch T, Shango M, Levine M, Brusovansky R, Akins R, Hayakawa K et al (2013) The relationship between the new taxonomy of *Streptococcus bovis* and its clonality to colon cancer, endocarditis, and biliary disease. Infection 41: 329–337. doi: 10.1007/s15010-012-0314-x 22886774 10.1007/s15010-012-0314-x

[5] Abdulamir AS, Hafidh RR, Bakar FA (2011) The association of *Streptococcus bovis/gallolyticus* with colorectal tumors: the nature and the underlying mechanisms of its etiological role. Journal of Experimental and Clinical Cancer Research 30: 11–11. doi: 10.1186/1756-9966-30-11 21247505 10.1186/1756-9966-30-11 PMC3032743

[6] Romero-Hernández B, Tedim AP, Sánchez-Herrero JF, Librado P, Rozas J, Muñoz G, et al (2015) *Streptococcus gallolyticus* subsp. *gallolyticus* from human and animal origins: genetic diversity, antimicrobial susceptibility, and characterization of a vancomycin-resistant calf isolate carrying a vanA-Tn1546-like element. Antimicrobial agents and chemotherapy 59: 2006–2015. doi: 10.1128/AAC.04083-14 25605355 10.1128/AAC.04083-14 PMC4356806

[7] Hinse D, Vollmer T, Rückert C, Blom J, Kalinowski J, Knabbe C, et al (2011) Complete genome and comparative analysis of *Streptococcus gallolyticus* subsp. *gallolyticus*, an emerging pathogen of infective endocarditis. BMC genomics 12: 400 doi: 10.1186/1471-2164-12-400 21824414 10.1186/1471-2164-12-400 PMC3173452

[8] Lin I-H, Liu T-T, Teng Y-T, Wu H-L, Liu Y-M, Wu K-M, et al (2011) Sequencing and comparative genome analysis of two pathogenic *Streptococcus gallolyticus* subspecies: genome plasticity, adaptation and virulence. PLoS One 6: e20519 doi: 10.1371/journal.pone.0020519 21633709 10.1371/journal.pone.0020519 PMC3102119

[9] Rusniok C, Couvé E, Da Cunha V, El Gana R, Zidane N, Bouchier C, et al (2010) Genome sequence of *Streptococcus gallolyticus*: insights into its adaptation to the bovine rumen and its ability to cause endocarditis. Journal of bacteriology 192: 2266–2276. doi: 10.1128/JB.01659-09 20139183 10.1128/JB.01659-09 PMC2849448

[10] Vollmer T, Hinse D, Kleesiek K, Dreier J (2010) Interactions between endocarditis-derived *Streptococcus gallolyticus* subsp. *gallolyticus* isolates and human endothelial cells. BMC Microbiol 10: 78 doi: 10.1186/1471-2180-10-78 20233397 10.1186/1471-2180-10-78 PMC2846920

[11] Aderem A (2003) Phagocytosis and the inflammatory response. J Infect Dis 187 Suppl 2: S340–5. 12792849 10.1086/374747

[12] Benoit M, Thuny F, Le Priol Y, Lepidi H, Bastonero S, Casalta J-P, et al (2010) The transcriptional programme of human heart valves reveals the natural history of infective endocarditis. PLoS ONE 5: e8939 doi: 10.1371/journal.pone.0008939 20126625 10.1371/journal.pone.0008939 PMC2812508

[13] Boleij A, Muytjens CMJ, Bukhari SI, Cayet N, Glaser P, Hermans PWM, et al (2011) Novel clues on the specific association of *Streptococcus gallolyticus* subsp *gallolyticus* with colorectal cancer. J Infect Dis 203: 1101–1109. doi: 10.1093/infdis/jiq169 21451000 10.1093/infdis/jiq169

[14] Underhill DM, Ozinsky A (2002) Phagocytosis of microbes: complexity in action. Annual review of immunology 20: 825–852. doi: 10.1146/annurev.immunol.20.103001.114744 11861619 10.1146/annurev.immunol.20.103001.114744

[15] Haas A (2007) The phagosome: compartment with a license to kill. Traffic 8: 311–330. doi: 10.1111/j.1600-0854.2006.00531.x 17274798 10.1111/j.1600-0854.2006.00531.x

[16] Nüsse O (2011) Biochemistry of the phagosome: the challenge to study a transient organelle. ScientificWorldJournal 11: 2364–2381. doi: 10.1100/2011/741046 22194668 10.1100/2011/741046 PMC3236389

[17] Smith LM, May RC (2013) Mechanisms of microbial escape from phagocyte killing. Biochemical Society Transactions 41: 475–490. doi: 10.1042/BST20130014 23514140 10.1042/BST20130014

[18] El Aamri F, Remuzgo-Martínez S, Acosta F, Real F, Ramos-Vivas J, Icardo JM, et al (2015) Interactions of *Streptococcus iniae* with phagocytic cell line. Microbes and Infection 17: 258–265. doi: 10.1016/j.micinf.2014.06.006 24956597 10.1016/j.micinf.2014.06.006

[19] De Herdt P, Haesebrouck F, Charlier G, Ducatelle R, Devriese LA, Vandenbossche G (1995) Intracellular survival and multiplication of virulent and less virulent strains of *Streptococcus bovis* in pigeon macrophages. Vet Microbiol 45: 157–169. 7571367 10.1016/0378-1135(95)00035-9

[20] Kaneko M, Emoto Y, Emoto M (2016) A Simple, Reproducible, Inexpensive, Yet Old-Fashioned Method for Determining Phagocytic and Bactericidal Activities of Macrophages. Yonsei medical journal 57: 283–290. doi: 10.3349/ymj.2016.57.2.283 26847277 10.3349/ymj.2016.57.2.283 PMC4740517

[21] Wang H, Joseph JA (1999) Quantifying cellular oxidative stress by dichlorofluorescein assay using microplate reader. Free Radic Biol Med 27: 612–616. 10490282 10.1016/s0891-5849(99)00107-0

[22] Vandesompele J, De Preter K, Pattyn F, Poppe B, Van Roy N, De Paepe A et al (2002) Accurate normalization of real-time quantitative RT-PCR data by geometric averaging of multiple internal control genes. Genome biology 3: 1. 10.1186/gb-2002-3-7-research0034 PMC126239 12184808

[23] Tsuchiya S, Yamabe M, Yamaguchi Y, Kobayashi Y, Konno T, Keiya T (1980) Establishment and characterization of a human acute monocytic leukemia cell line (THP-1). International journal of cancer 26: 171–176. 6970727 10.1002/ijc.2910260208

[24] Mouithys-Mickalad A, Deby-Dupont G, Dogne J-M, de Leval X, Kohnen S, Navet R, et al (2004) Effects of COX-2 inhibitors on ROS produced by Chlamydia pneumoniae-primed human promonocytic cells (THP-1). Biochemical and biophysical research communications 325: 1122–1130. doi: 10.1016/j.bbrc.2004.10.155 15555544 10.1016/j.bbrc.2004.10.155

[25] Song X-M, Connor W, Hokamp K, Babiuk LA, Potter AA (2009) Transcriptome studies on *Streptococcus pneumoniae*, illustration of early response genes to THP-1 human macrophages. Genomics 93: 72–82. doi: 10.1016/j.ygeno.2008.09.008 18848982 10.1016/j.ygeno.2008.09.008

[26] Srisuwan S, Tongtawe P, Srimanote P, Voravuthikunchai SP (2014) Rhodomyrtone modulates innate immune responses of THP-1 monocytes to assist in clearing methicillin-resistant *Staphylococcus aureu*s. PloS one 9: e110321 doi: 10.1371/journal.pone.0110321 25329066 10.1371/journal.pone.0110321 PMC4199624

[27] Masoudian M, Derakhshandeh A, Seno MG (2015) *Brucella melitensis* and *Mycobacterium tuberculosis* depict overlapping gene expression patterns induced in infected THP-1 macrophages. Iranian journal of veterinary research 16: 368 27175205 PMC4782677

[28] Kohro T, Tanaka T, Murakami T, Wada Y, Aburatani H, Hamakubo T, et al (2004) A comparison of differences in the gene expression profiles of phorbol 12-myristate 13-acetate differentiated THP-1 cells and human monocyte-derived macrophage. Journal of atherosclerosis and thrombosis 11: 88–97. 15153668 10.5551/jat.11.88

[29] Rosenberger CM, Finlay BB (2003) Phagocyte sabotage: disruption of macrophage signalling by bacterial pathogens. Nature reviews Molecular cell biology 4: 385–396. doi: 10.1038/nrm1104 12728272 10.1038/nrm1104

[30] Cotter PD, Hill C (2003) Surviving the acid test: responses of gram-positive bacteria to low pH. Microbiology and Molecular Biology Reviews 67: 429–453. doi: 10.1128/MMBR.67.3.429-453.2003 12966143 10.1128/MMBR.67.3.429-453.2003 PMC193868

[31] Dupré-Crochet S, Erard M, Nüβe O (2013) ROS production in phagocytes: why, when, and where? J Leukoc Biol 94: 657–670. doi: 10.1189/jlb.1012544 23610146 10.1189/jlb.1012544

[32] Davis KM, Weiser JN (2011) Modifications to the peptidoglycan backbone help bacteria to establish infection. Infection and immunity 79: 562–570. doi: 10.1128/IAI.00651-10 21041496 10.1128/IAI.00651-10 PMC3028845

[33] Kurtovic A, Jarvis GN, Mantovani HC, Russell JB (2003) Ability of lysozyme and 2-deoxyglucose to differentiate human and bovine *Streptococcus bovis* strains. Journal of clinical microbiology 41: 3951–3954. doi: 10.1128/JCM.41.8.3951-3954.2003 12904427 10.1128/JCM.41.8.3951-3954.2003 PMC179835

[34] Baker J, Derr A, Karuppaiah K, MacGilvray M, Kajfasz J, Faustoferri RC, et al (2014) S*treptococcus mutans* NADH oxidase lies at the intersection of overlapping regulons controlled by oxygen and NAD^+^ levels. Journal of bacteriology 196: 2166–2177. doi: 10.1128/JB.01542-14 24682329 10.1128/JB.01542-14 PMC4054193

[35] Reichmann NT, Cassona CP, Gründling A (2013) Revised mechanism of D-alanine incorporation into cell wall polymers in Gram-positive bacteria. Microbiology 159: 1868–1877. doi: 10.1099/mic.0.069898-0 23858088 10.1099/mic.0.069898-0 PMC3783018

[36] Poyart C, Pellegrini E, Marceau M, Baptista M, Jaubert F, Lamy M-C, et al (2003) Attenuated virulence of *Streptococcus agalactiae* deficient in D-alanyl-lipoteichoic acid is due to an increased susceptibility to defensins and phagocytic cells. Molecular microbiology 49: 1615–1625. 12950925 10.1046/j.1365-2958.2003.03655.x

[37] Deutscher J, Francke C, Postma PW (2006) How phosphotransferase system-related protein phosphorylation regulates carbohydrate metabolism in bacteria. Microbiology and Molecular Biology Reviews 70: 939–1031. doi: 10.1128/MMBR.00024-06 17158705 10.1128/MMBR.00024-06 PMC1698508

[38] Jung C-J, Zheng Q-H, Shieh Y-H, Lin C-S, Chia J-S (2009) *Streptococcus mutans* autolysin AtlA is a fibronectin-binding protein and contributes to bacterial survival in the bloodstream and virulence for infective endocarditis. Molecular microbiology 74: 888–902. doi: 10.1111/j.1365-2958.2009.06903.x 19818020 10.1111/j.1365-2958.2009.06903.x

