# Supplementary material for: Transcriptome analysis of Streptococcus gallolyticus subsp. gallolyticus in interaction with THP-1 macrophage-like cells
Source: PLoS One. 2017 Jul 3;12(7):e0180044. doi: 10.1371/journal.pone.0180044 (PMC5495212; doi:10.1371/journal.pone.0180044)
Supplement: S1 File — Supplement File S1: Supplementary methods, figures (A-C) and tables (A+B). (DOCX) [file pone.0180044.s001.docx]

**Supplement File S1: Supplementary methods, figures (A-C) and tables (A+B)**

**Methods: Microsopic analysis**

Bacterial cells (overnight culture) were labeled in DPBS with 0.1 mg/ml fluorescein isothiocyanate (Thermo Scientific, Waltham, USA) for 30 min at room temperature. After incubation, the bacterial cells were centrifuged (5000 × *g*, 5 min), washed thrice and the resulting pellet was suspended in DPBS supplemented with 0.5 mg/ml sulfo-NHS-LC-biotin (Thermo Scientific, Waltham, USA) for 30 min at 37 °C. After washing the bacterial suspension three times with DPBS, phagocytosis assay was carried out as described above. At time point 1 h, macrophages with labeled bacteria were fixed with 4 % formaldehyde for 15 min at room temperature and, subsequently, nonspecific binding sites were blocked with 5 % FCS in DPBS for 5 min. Extracellular bacteria were labeled with Streptavidin-Alexa Fluor 546 (Thermo Scientific, Waltham, USA) for 45 min at room temperature. All labeling was also performed alone as control to confirm that to shine in other channels. Microscopy was performed with the Nikon Eclipse TE2000-S (Nikon instruments, Düsseldorf, Germany) and the images were processed and merged with ImageJ software (NIH, USA). At least 100 macrophages were counted (set as 1). Number of bacterial cells (intracellular or extracellular) were divided through the number of macrophages (bacterial cells per macrophage; see Table 2).

**Figure A**


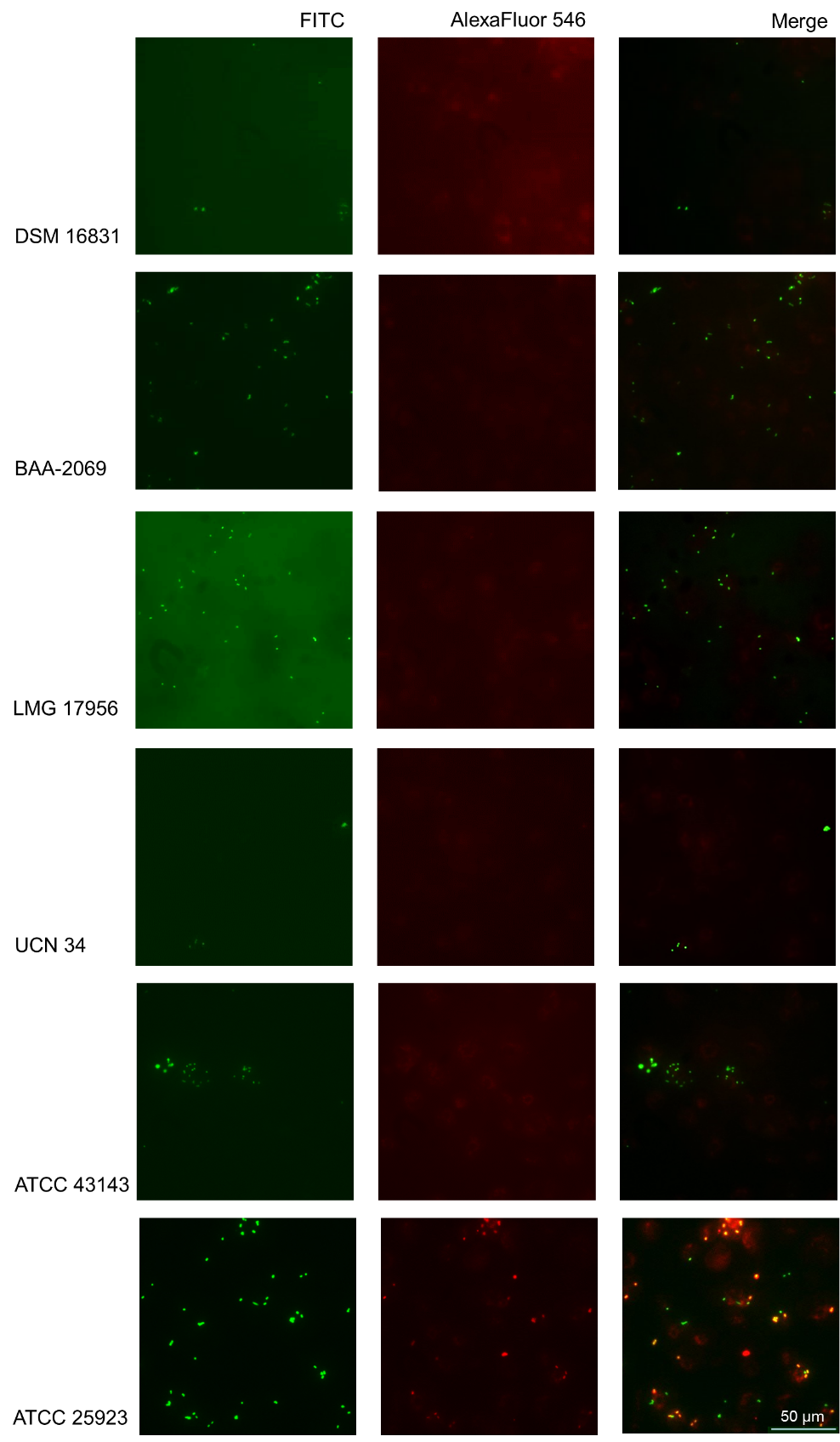


**Figure A: Microscopic pictures - bacteria labeled with FITC (green; all bacteria) and AlexaFluor546 (red; extracellular located bacteria).** It showed the localization of the bacteria with THP-1 macrophages 1 h after enabled phagocytosis. The magnification is 400x.

**Figure B**

**Figure B: Relative quantification of intracellular reactive oxygen species and cytotoxicity of bacteria to macrophages**

1) Emission of intracellular DCF after stimulation of THP-1 macrophages with different *S. gallolyticus* subsp. *gallolyticus* strains in comparison to the *S. aureus* strain ATCC25923 and H_2_O_2_ (1 mM) at time point 0 h.

2) Lysis of THP-1 macrophages by different *S. gallolyticus* subsp. *gallolyticus* strains and the *S. aureus* strain ATCC 25923 measured with a LDH cytotoxicity assay (t = 5 h) in comparison to the lysis of all THP-1 macrophages (100 %).

Shown is the mean with standard error. Results of statistical analysis between strains or control (Mann-Whitney U test): *: p < 0.05; **: p < 0.005; n = 3

**Figure C**

**Figure C: Relative gene expression of *IL1B* (1), *IL6* (2) and *IL8* (3) of THP-1 macrophages after phagocytosis of different *S. gallolyticus* subsp. *gallolyticus* strains in comparison to the *S. aureus* strain ATCC 25923**. Relative gene expression of THP-1 macrophages 5 h after phagocytosis of different bacterial cells compared to control (black bar, set as 1, see dotted line). Shown is the mean with standard error; n = 4; NC = negative control

**Tables:**

Table A: **Oligonucleotides used for relative quantitative real-time PCR analysis**

| Human | | |
| --- | --- | --- |
| Product name (gene name) | Annealing temperature | 5′–3′ sequence: Forward  Reverse |
| Ribosomal protein L13 (*RPL13A*) | 63°C | CGGAAGGTGGTGGTCGTA  CTCGGGAAGGGTTGGTGT |
| Hydroxymethylbilane synthase (HMBS) | 63°C | CTGCCAGAGAAGAGTGTG  AGCTGTTGCCAGGATGAT |
| Succinate dehydrogenase complex, subunit A, flavoprotein (*SDHA*) | 63°C | AACTCGCTCTTGGACCTG  GAGTCGCAGTTCCGATGT |
| Interleukin-1β (*IL-1B*) | 63°C | ACAGATGAAGTGCTCCTTCCA  GTCGGAGATTCGTAGCTGGAT |
| Interleukin-6 (*IL6*) | 63°C | ACAGCCACTCACCTCTTCAG  GTGCCTCTTTGCTGCTTTCAC |
| Interleukin-8 (*IL8*) | 63°C | GAACTGAGAGTGATTGAGAGTGGA  CTCTTCAAAAACTTCTCCACAACC |
| *S. gallolyticus* subsp. *gallolyticus* | | |
| Product name (gene name) | Annealing temperature | 5′–3′ sequence: Forward  Reverse |
| 16S ribosomal RNA (*16S*) | 65°C | TTATGACCTGGGCTACAC  CCTACAATCCGAACTGAG |
| 23S ribosomal RNA (*23S*) | 65°C | CTKCCAAGAAAAGCYTCT  CATTTTGCCDAGTTCCTT |
| Autolysin (*SGGBAA_c13580*) | 65°C | ATCGTCCTGCCCTTTATC  CGGTTTCAGTTGCCAATC |
| carbonic anhydrase (*SGGBAA-c20810*) | 65°C | TCAGCTTGAGGCAGAGCTAC  GCGGACGGCAAACAGATT |
| D-alanyl-D-alanine carrier protein ligase (*dltA*) | 65°C | GACGGCATCATGTAATCC  TATCACACGGGTGACTTAGG |
| D-alanine transfer from Dcp to undecaprenol-phosphate (*dltB*) | 65°C | AGCTGTGGCAATAGCAAGCTAC  ACGGCGTGCTGAGTGAATTTA |
| D-alanine carrier protein (*dltC*) | 65°C | AGCATGGCAAAGTTCCTC  ACGGTGGAACTCATTGTG |
| D-alanine transfer from undecaprenol-phosphate to the poly(glycerophosphate) chain (*dltD*) | 65°C | CCACCGAGTTTGCTGTAAG  GTGTTTATGACCGCTACGC |
| glycogen synthase (*glgA*) | 65°C | AAGTAGGATGGCGTCATCAG  ACGGAATCATGGCAGTATGG |
| 1,4-alpha-glucan branching enzyme (*glgB*) | 65°C | CAGGACGATTACGGAAAC  GTTTGCGTGAAGTGCTTG |
| glucose-1-phosphate adenylyltransferase (*glgC*) | 65°C | GTTTGCGTGAAGTGCTTG  TTTGGTGGTGCGATAAGG |
| glucose-1-phosphate adenylyltransferase (*glgD*) | 65°C | GACGGAACAGGCAAAGAAG  CCAGAGCGTTTCAGGTAAG |
| glycogen phosphorylase (*glgP*) | 65°C | CTGCCGAGTTTATGCGTCTGC  TTCTCCAGCCACTGCCAATCC |
| nucleoside diphosphate kinase (*ndk*) | 65°C | CAAACCAGAGGGCGATTTC  GAGGTGCTTCACCGTATTG |
| acyl-acyl carrier protein (ACP):phosphate acyltransferase (*plsX*) | 65°C | GCTATGGGTGGCGACAATG  AGCCCTGGACGGTCAATAC |
| post-translocation molecular chaperone (*prsA1*) | 65°C | TATCGGTGCTGGTGTAAC  GTGCGCTTGAGAATGATG |
| 2,3,4,5-tetrahydropyridine-2,6-dicarboxylate N-succinyltransferase (*dapD*) | 65°C | TTACGCAAAGCGTCTTCGAGTG  TTGACATGGGTGCTGTTCTTGG |
| Phosphopentomutase (*drm*) | 65°C | AAATCGCAGCACACGAAG  CATTACGACGGTGACCATAC |
| bifunctional N-acetylglucosamine-1-phosphate (*gcaD*) | 65°C | TGAATCTGGGCGAACATGG  ACTTGCGACAGCAGAAGAC |
| PTS system N-acetylgalactosamine-specific transporter subunit IIA (*levA*) | 65°C | TTGGTGGTAGCCCACTTACAAC  TGAAGAGCTGCTTGAGCTTCTG |
| NADH oxidase (*nox*) | 65°C | CCTTAATTGGCGGGATGATTGG  CGGTTGTGGTATGGCATTATGG |

**Table B:** **Statistical results between strains in their survival at different time points (Figure 1).** Statistical significances were calculated with the Mann-Whitney U test: *: p < 0.05; **: p < 0.005; ***: p < 0.0005; ****: p < 0.0001; ns = not significant; - = same sample; tp = time point.

|  | tp (h) | DSM16831 | BAA-2069 | LMG17956 | UCN34 | ATCC43143 | SA |
| --- | --- | --- | --- | --- | --- | --- | --- |
| DSM16831 | 2.5 | - | **** | ns | ns | * | *** |
|  | 5 | - | *** | *** | ** | ** | *** |
|  | 8 | - | **** | **** | **** | *** | **** |
| BAA-2069 | 2.5 | **** | - | ** | ** | ns | ns |
|  | 5 | *** | - | ns | ns | ns | ns |
|  | 8 | **** | - | **** | ns | ns | ns |
| LMG17956 | 2.5 | ns | ** | - | ns | ns | ** |
|  | 5 | *** | ns | - | ns | ns | * |
|  | 8 | **** | **** | - | ns | * | **** |
| UCN 34 | 2.5 | ns | ** | ns | - | ns | ** |
|  | 5 | ** | ns | ns | - | ns | * |
|  | 8 | **** | ns | ns | - | ns | ** |
| ATCC43143 | 2.5 | * | ns | ns | ns | - | * |
|  | 5 | ** | ns | ns | ns | - | * |
|  | 8 | *** | ns | * | ns | - | ** |
| SA | 2.5 | *** | ns | ** | ** | * | - |
|  | 5 | *** | ns | * | * | * | - |
|  | 8 | **** | ns | **** | ** | ** | - |
